# Survey on Psychotherapy training In Ukraine, and its importance in the current war.

**DOI:** 10.1192/j.eurpsy.2023.204

**Published:** 2023-07-19

**Authors:** K. Shalak

**Affiliations:** Lviv Regional Clinical Hospital, Lviv, Ukraine

## Abstract

**Abstract:**

Psychiatrists postgraduate training in Ukraine lasts only for 18 months and the curriculum includes psychotherapy. However, little is known about its quality and provision. The aims of the research were to investigate the current psychotherapy training status in Ukraine, to examine how it is included in psychiatry training and to identify access to it. An anonymous online survey was conducted among early career psychiatrists (psychiatry trainees or psychiatrists within 5 years after specialising in psychiatry). The findings show that the minority had access to psychotherapy training though the majority of responders consider it to be an important part of psychiatry training. This study encourages to improve the accessibility and quality of psychotherapy training in Ukraine. It shows that despite the low accessibility of psychotherapy training for psychiatry trainees there is a high need in it. It is even more relevant in times of war, given its negative consequences for mental health and the growing need for educated psychotherapists.

**Disclosure of Interest:**

None Declared

